# Supplementary material for: Focal control of non-invasive deep brain stimulation using multipolar temporal interference
Source: Bioelectron Med. 2025 Mar 27;11:7. doi: 10.1186/s42234-025-00169-6 (PMC11948895; doi:10.1186/s42234-025-00169-6)
Supplement: Supplementary file 1 — Supplementary Material 1. Supplementary Figure 1. Phase interactions in mTI.Two envelopes, in phase, create a larger aggregate envelope.Two envelopes, with offset phase, create an poorly defined aggregate envelope.Two envelopes, with 180 degree phase offset, create no aggregate envelope. It is important to ensure phase alignment at the deep brain target. In this study, we ensured phase alignment as the aggregate envelope signal could be visually confirmed to be in phase using the recording electrode at the brain target. In future work, if no recording electrode is present at the target, a strategy to apply stimulation which provides in phase envelopes at the deep brain target is needed. Supplementary Figure 2: Example of functionally-related neural activity recorded from the SC Spiking activity of the unit shown in Fig. 4A of the main manuscript, focusing here on the activity following visual target onset. Top left panel shows eye movement trajectories for three 12 deg saccade vectors directed contralateral to the side of SC recording, with the color scheme denoting different vertical components. Middle to bottom left panels show, using the same color scheme, the horizontal eye position traces, the rasters of neural activity recorded from a channel in the intermediate layers of the SC, highlighted in red in the middle column, and the associated spike density functions. Middle column shows spike density functions for those channels positioned within the intermediate layers of the SC [file 42234_2025_169_MOESM1_ESM.docx]

**
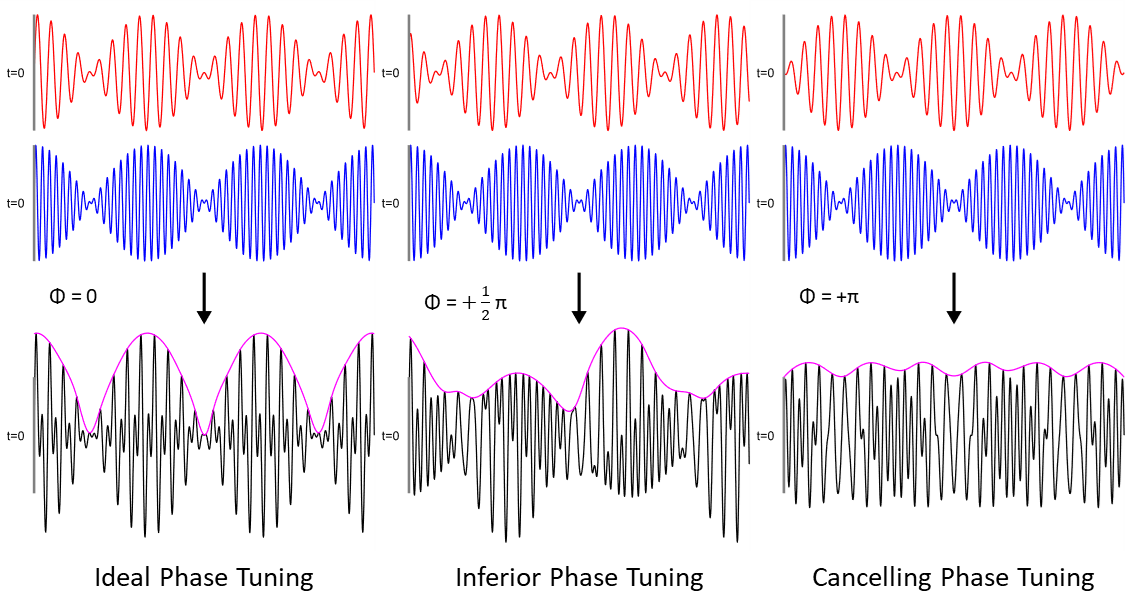
**

**Supplementary Figure 1. Phase interactions in mTI**

(left panel) Two envelopes, in phase, create a larger aggregate envelope. (middle panel) Two envelopes, with offset phase, create an poorly defined aggregate envelope. (right panel) Two envelopes, with 180 degree phase offset, create no aggregate envelope. It is important to ensure phase alignment at the deep brain target. In this study, we ensured phase alignment as the aggregate envelope signal could be visually confirmed to be in phase using the recording electrode at the brain target. In future work, if no recording electrode is present at the target, a strategy to apply stimulation which provides in phase envelopes at the deep brain target is needed.


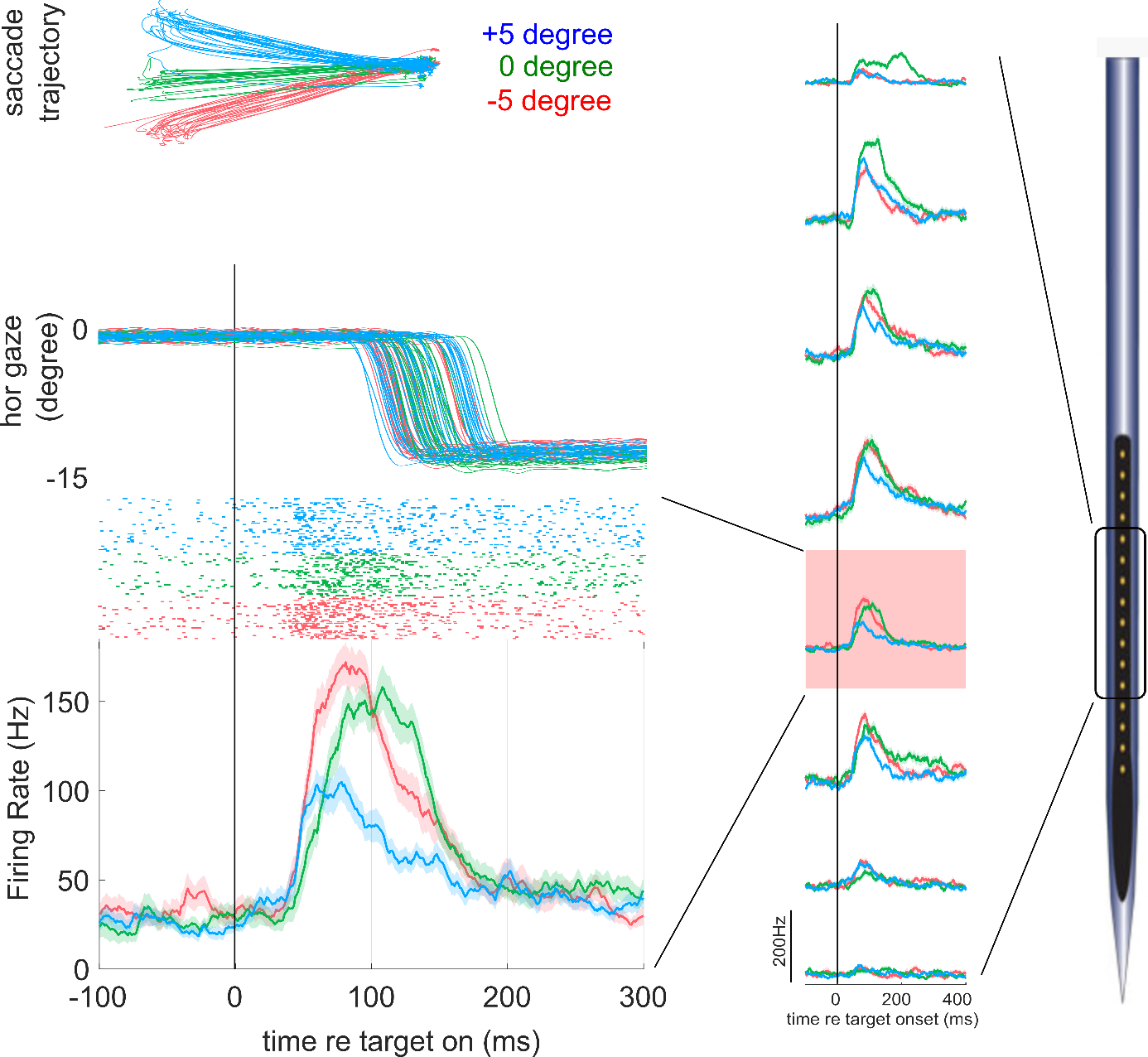


**Supplementary Figure 2: Example of functionally-related neural activity recorded from the SC** Spiking activity of the unit shown in Fig. 4A of the main manuscript, focusing here on the activity following visual target onset. Top left panel shows eye movement trajectories for three 12 deg saccade vectors directed contralateral to the side of SC recording, with the color scheme denoting different vertical components. Middle to bottom left panels show, using the same color scheme, the horizontal eye position traces (downward deflections denote left movements), the rasters of neural activity recorded from a channel in the intermediate layers of the SC, highlighted in red in the middle column, and the associated spike density functions. Middle column shows spike density functions for those channels positioned within the intermediate layers of the SC.
